# Service-Learning and Chinese College Students' Knowledge Transfer Development

**DOI:** 10.3389/fpsyg.2020.606334

**Published:** 2020-12-14

**Authors:** Cong Wang, Wenfan Yan, Fangfang Guo, Yulan Li, Meilin Yao

**Affiliations:** ^1^Faculty of Psychology, Beijing Normal University, Beijing, China; ^2^Department of Leadership in Education, College of Education and Human Development, University of Massachusetts, Boston, MA, United States; ^3^School-based Mental Health Center, Beijing Information Science and Technology University, Beijing, China; ^4^School of Education Science, Nanning Normal University, Nanning, China

**Keywords:** psychological control, higher education, knowledge transfer, service-learning, mastery goal orientation

## Abstract

As a form of experiential education, service learning (SL) shows great potential for promoting students' knowledge transfer as it offers students opportunities to apply what they have learned in classrooms to serve communities in real-life contexts. To explore how students' knowledge transfer evolves during SL, we collected longitudinal survey data from 96 Chinese college students in a 9-week SL program. Results indicate that (a) students' perceived knowledge transfer in SL did not follow a linear trajectory. Although students' perceived knowledge transfer at the end of SL was significantly higher than those at the beginning, a slight drop was observed in the middle of SL; (b) the developmental pattern of perceived knowledge transfer varied across students; and (c) students' perceived knowledge transfer development during SL was associated with mastery goal orientation and perceptions of psychologically controlling behaviors from their SL supervisors. By providing evidence of the dynamic process and mechanisms of students' knowledge transfer development, the present study adds to our understanding of how, when, and why the benefits of SL are realized.

## 1. Introduction

Knowledge transfer, the ability to apply knowledge and skills learned in school into a new situation, is an important indicator of educational success (Bransford and Schwartz, 1999; Wang et al., 2020), particularly for college students, as most of them will enter the workforce after graduation and will be expected from their employers to apply what they have learned in college into authentic situations. Promoting students' deep learning and knowledge transferability is especially critical for the Chinese education system, as it has been criticized for emphasizing too much on helping students achieve good exam scores in standardized tests (Guo-Brennan, 2016) and impeding students' abilities to transfer what they have learned in class to real-life situations (Guo et al., 2016a). Service learning (SL), a pedagogical method that combines academic learning and community service, may have great potential for promoting students' knowledge transfer because it offers students opportunities to apply what they have learned in classrooms to serve communities in real-life contexts (Wang et al., 2019b). It has been advocated for decades in Western countries (Furco et al., 2016) and considered one of the high-impact educational practices in higher education (Kuh, 2008); however, to promote SL in contemporary Chinese education, more empirical evidence that supports the impact of SL on Chinese students' outcomes is needed.

Mounting evidence suggests that SL benefits college students' academically, professionally, and personally (Eyler and Giles, 1999; Knapp et al., 2010; Yorio and Ye, 2012; Bringle et al., 2016; Furco et al., 2016). Nevertheless, mixed findings have also been reported regarding the academic benefits of SL for students (Furco et al., 2016; Song et al., 2017). Recently, more attention has been directed toward the dynamic processes and mechanisms of students' development during SL (Li et al., 2016; Guo et al., 2016b), as these variables are critical to better understand how SL works, when SL is effective, and who SL benefits, especially as SL experiences are increasing in number across institutions of higher education (Furco et al., 2016). Using a case study approach, Guo et al. (2016b) found that college students' behavioral, emotional, and cognitive engagement fluctuated over the 9-week SL program. Based on the characteristics of students' engagement development, they divided the whole SL into four developmental stages: confusion and hesitancy, enlightenment and enthusiasm, fluctuation and adjustment, and stabilization and routinization.

While a growing body of research has shown the positive impact of SL on college students' knowledge transfer (Markus et al., 1993; Eyler and Giles, 1999; Deeley, 2010; Prentice and Robinson, 2010; Gerholz et al., 2018; Wang et al., 2019b), data about the dynamic processes of knowledge transfer development during SL have not yet been documented. This study therefore set out to investigate the development characteristics of college students' knowledge transfer within the context of a 9-week SL program. We designed a longitudinal study to track students in SL by measuring their knowledge transfer at eight time points. Previous research suggests that college students' self-reports of knowledge transfer can provide valuable information about their knowledge transferability (Wang et al., 2020). Several studies have shown that college students' perceived knowledge transfer is positively associated with their perceived learning and course grades (Hsu et al., 2019; Wang et al., 2019a). Because assessing actual transfer performance at multiple times costs researchers' laborious hours, in the current research, we studied college students' perceived knowledge transfer instead of their actual knowledge transfer.

In addition to examining the dynamic process of college students' knowledge transfer during SL, factors that influence the development characteristics of knowledge transfer are also important. While there are a number of perspectives to view student knowledge transfer development, the current study focuses on mastery goal orientation and perception of psychological control, as these two factors are well-grounded in the literature of education as consistent predictors of educational success (Kaplan and Maehr, 2007; Senko et al., 2011; Soenens et al., 2012; Ryan and Deci, 2017). Students with mastery goals orientation focus on acquiring and developing competence (Senko et al., 2011). Previous research has shown that students with mastery goal orientation performed better on a transfer task than the ones with performance goals (Bereby-Meyer and Kaplan, 2005; Belenky and Nokes-Malach, 2012). For instance, Belenky and Nokes-Malach (2012) studied 104 undergraduates to investigate how students' achievement goals interact with different forms of instruction to enhance transfer. They found a positive impact of mastery goal orientation on transfer. Students with mastery goal orientation are more likely to adopt deep learning strategies to process the learning materials, which may promote their knowledge transferability. The second factor, perception of psychological control, is grounded in self-determination theory (Deci and Ryan, 1985; Ryan and Deci, 2017). In the current study, perception of psychological control refers to the extent to which students perceive intrusive behaviors that pressure them to act, think, and feel in particular ways from their SL supervisors (Soenens et al., 2012). A correlational study conducted by Soenens et al. (2012) has shown that higher perceptions of psychological control were associated with lower metacognitive self-regulation and academic achievement. To date, the detrimental effects of psychologically controlling teaching on students' outcomes have been well-documented (Soenens et al., 2012; Haerens et al., 2015; Bartholomew et al., 2018), and in this study, we will explore the role of psychological control toward knowledge transfer development in an SL context.

The present investigation focused on three key research questions (RQs). First, how do students' perceived knowledge transfer change during a 9-week SL program (RQ1)? Second, are there different developmental patterns of perceived knowledge transfer across students (RQ2)? Third, we asked what factors affected students' perceived knowledge transfer development (RQ3)? The first and second research questions are exploratory in nature. Since students' engagement varies across developmental stages of SL (Li et al., 2016; Guo et al., 2016b), we expect to see a fluctuation in students' perceived knowledge transfer in the current study. With different motivation, personalities, and prior experiences, students may also demonstrate different trajectories in perceived knowledge transfer over the SL program of 9 weeks. For RQ3, based on the literature we reviewed, we expected that mastery goal orientation would facilitate students' perceived knowledge transfer development in SL, while perceptions of psychological control would hinder the process.

## 2. Materials and Methods

### 2.1. Participants

Participants in the study were undergraduates at a leading research university in China. This university is well-known for teacher education, education science, and basic learning in arts and sciences. We recruited participants from a psychology course entitled *Psychology of Learning*. The course is about fundamental concepts and empirical research findings related to learning sciences. Students who enrolled in the course were contacted at the beginning of the semester and invited to participate in a 9-week SL program embedded in the course. Out of the 111 students enrolled in the course, 96 students (75 females and 21 males) consented to participate in the research. All participants were sophomore students from the Department of Psychology. The research procedures and student surveys were approved by the institution's ethical committee.

### 2.2. Procedures and Measures

Students learned various learning principles (e.g., applied behavior analysis, conditioning theory, and learned helplessness) in their regular classroom learning. During weekends, they worked in a group of four to interact with children with special needs. Although the overall goal of the SL activity was “applying the knowledge and skills learned from the course of Psychology of Learning to serve children with special needs,” the specific SL goals and activities were determined by the undergraduates themselves and might vary across groups. For instance, one group may focus on teaching the child to express his/her needs using appropriate words, while the other group may aim to teach the child to pass and catch a ball. Each service group had a supervisor who provided support during and after SL activities. Students wrote reflective journal entries immediately after each service activity. Students also completed a series of questionnaires prior to and after SL regarding themselves and their SL experiences.

#### 2.2.1. Perceived Knowledge Transfer

To investigate the development of knowledge transfer during SL, students' perceived knowledge transfer was assessed at eight time points. We asked students to report their levels of knowledge transfer in their weekly reflective journal entries. A single-item measure was used (“Please rate to what extent you applied the knowledge you've learned into this week's SL activity”), with the scale ranging from 0 “none” to 4 “a lot.” To ensure the validity of the self-report item, the first author went over students' reflective journal entries and found that students who had high scores (i.e., 3 or 4) on this item used more psychology terms and concepts in their reflective journal entries. Furthermore, the sum of the eight perceived knowledge transfer scores was positively associated with the overall perceived knowledge transfer score in their post-SL reflective journal entries (*r* = 0.51, *p* <0.001).

#### 2.2.2. Mastery Goal Orientation

In the pre-SL questionnaire, we used the six-item Task Goal Orientation Scale (Midgley et al., 1998) to assess students' goal to develop their understanding and skills. The items were translated into Chinese and rated on a 5-point scale (1 = not at all true of me, 5 = very true of me). Sample items included: “I like school work that I'll learn from, even if I make a lot of mistakes.” Internal consistency reliability was acceptable (Cronbach's alpha coefficient = 0.71).

#### 2.2.3. Perception of Psychological Control

In the post-SL questionnaire, we assessed students' perceptions of psychologically controlling teaching behaviors from their supervisors using the seven-item Psychologically Controlling Teaching Scale (Soenens et al., 2012). We translated the items into Chinese and adapted to the service-learning context. Higher scores on the scale reflect a more controlling supervising style. Example items include the following: “My supervisor often interrupts me,” and “My supervisor is less friendly with me if I do not see things his/her way.” Scale points ranged from 1 “completely disagree” to 5 “completely agree.” Internal consistency reliability was good (Cronbach's alpha coefficient = 0.93).

### 2.3. Analysis

To explore how students' knowledge transfer evolves during SL (RQ1), we assessed their perceived knowledge transfer across eight time points from Week 6 to 14 in an 18-week semester. Data from previous studies suggest that there are four developmental stages of student engagement during a 9-week SL program (Guo et al., 2016b). Descriptive statistics of student perceived knowledge transfer across eight time points confirms such stage classification. Therefore, we described the development of student perceived knowledge transfer using the four stages that identified from previous work, namely the confusion and hesitancy stage (1st time), the enlightenment and enthusiasm stage (2nd and 3rd time), the fluctuation and adjustment stage (4th to 7th time), and the stabilization and routinization stage (8th time). Repeated-measures analysis of variance (ANOVA) was used to examine the fluctuations of perceived knowledge transfer across the four developmental stages. To further understand the pattern of students' perceived knowledge transfer development across students (RQ2), we conducted a model-based cluster analysis on students' perceived knowledge transfer across four developmental stages. The Bayesian information criteria (BIC) were considered to determine the optimal classification. After identifying the groupings of participants, repeated-measures ANOVA was conducted to test the development of perceived knowledge transfer across groups. To explore the differences between groups of students (RQ3), we conducted multinomial logistic regression to test the associations between the predictors (i.e., mastery goal orientation and psychological control) and the identified groups. Statistics were done using R version 4.0.2, the mclust (Scrucca et al., 2016), the nnet (Ripley et al., 2016), and the rstatix (Kassambara, 2020) packages.

## 3. Results

### 3.1. Students' Perceived Knowledge Transfer Fluctuated Across Four Stages of SL

**Figure 1:**
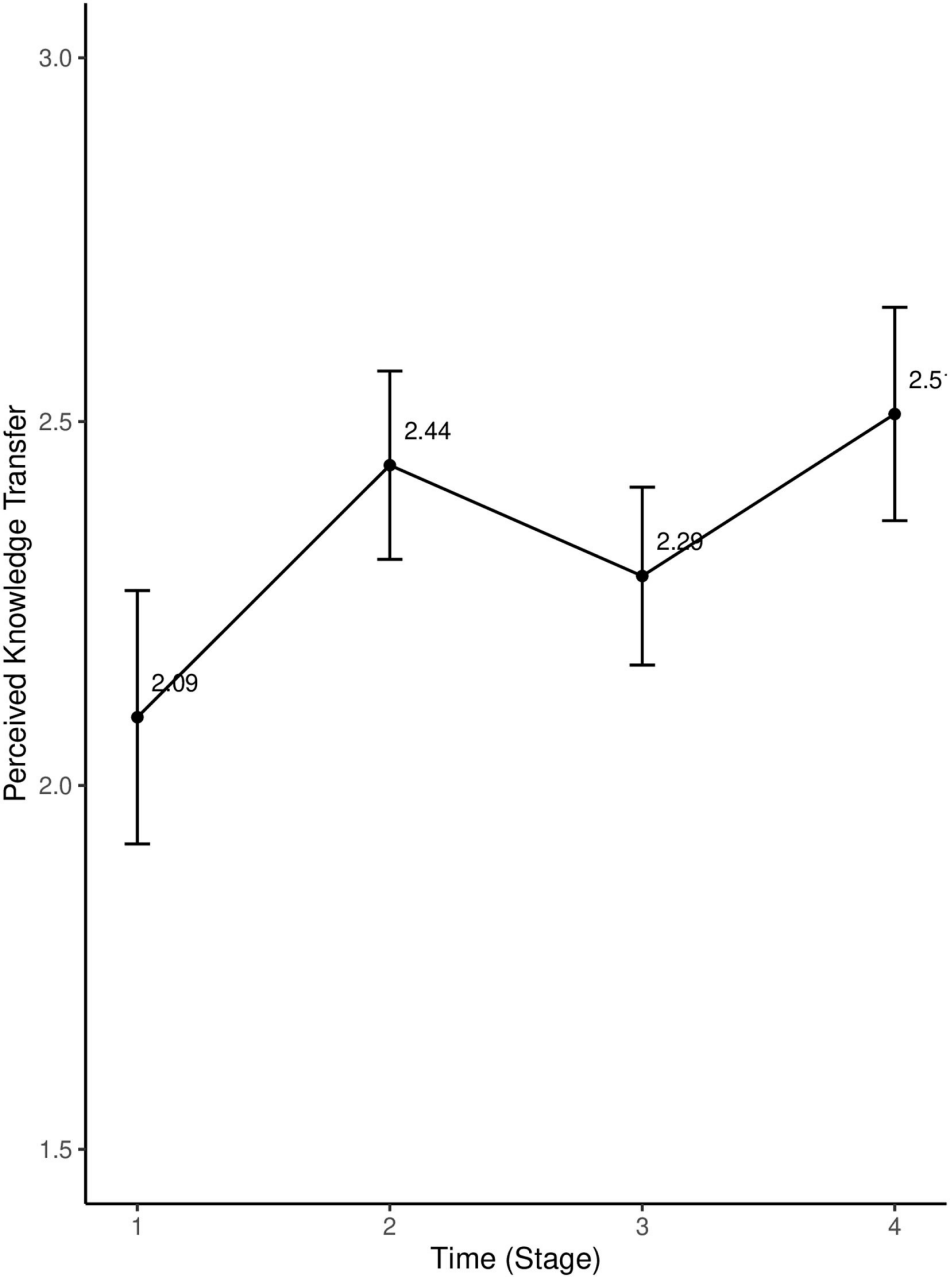
Trends in students' perceived knowledge transfer during a 9-week service learning (SL) program. Error bars represent 95% confidence intervals.

As shown in Figure 1, students' perceived knowledge transfer fluctuated across the whole SL program. Repeated measures ANOVA indicated significant differences in students' perceived knowledge transfer across the four stages [*F*_(2.6, 246.98)_ = 8.82, *p* <0.05, η^2^ = 0.05]. Pairwise comparisons suggested that students' perceived knowledge transfer significantly increased from Stage 1 to Stage 2 (*p* <0.001). No significant difference was found between Stages 2 and 3, although we observed a slight drop in the level of perceived knowledge transfer during Stage 3. students' perceived knowledge transfer rose to a high point and peaked during the last stage of the 9-week SL program. students' perceived knowledge transfer during Stage 4 was significantly higher than those in Stage 1 (*p* <0.001), suggesting that participating in the 9-week SL program might foster students' perceived knowledge transfer.

### 3.2. The Pattern of Perceived Knowledge Transfer Development Varied Across Students

**Figure 2:**
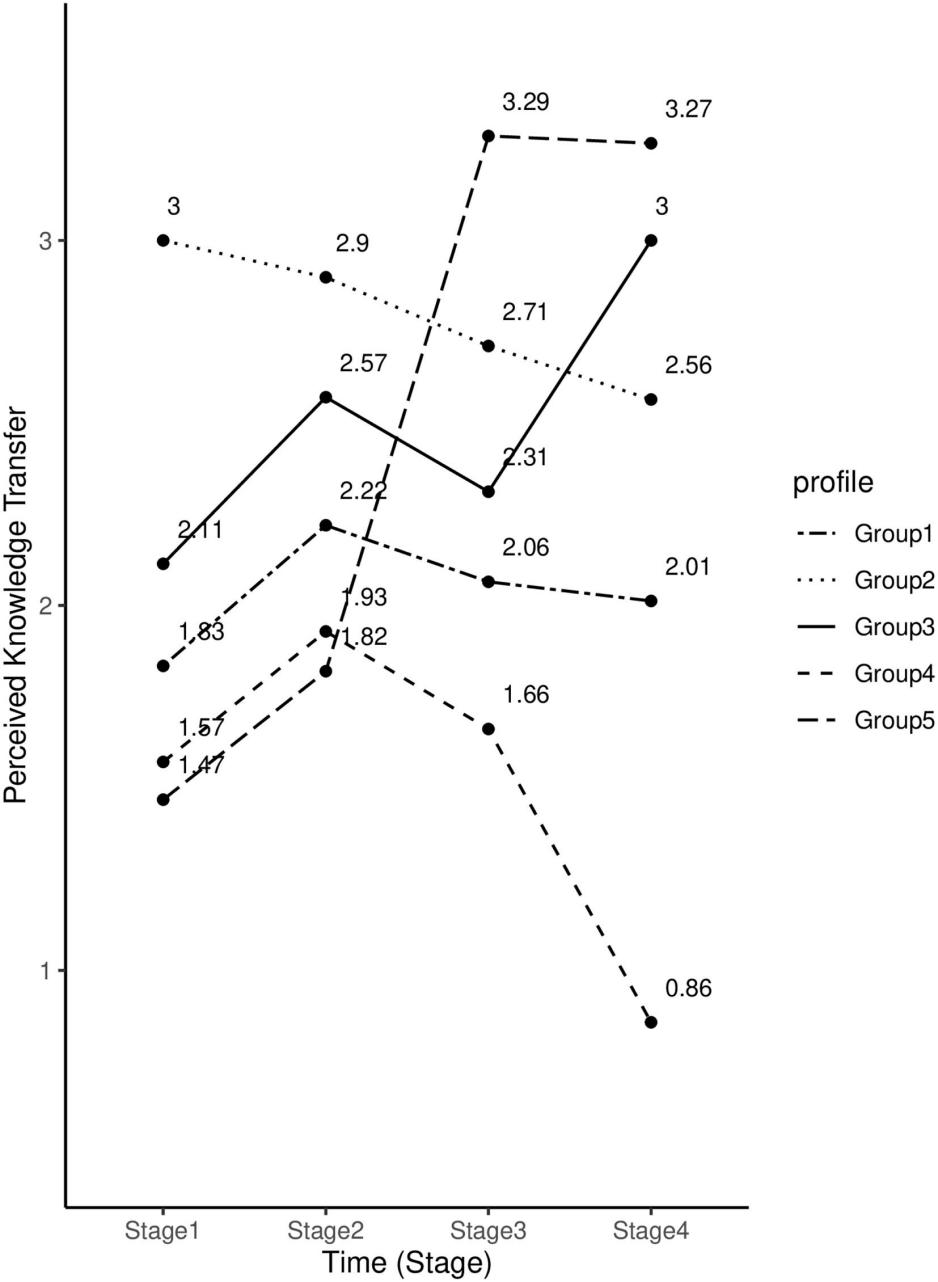
Trends in students' perceived knowledge transfer for different groups of students.

Students' perceived knowledge transfer scores across four stages were used in model-based cluster analysis for the categorization of groupings. Based on the best BIC values, the cluster analysis approach produced five clusters with 27 students in Group 1, 12 students in Group 2, 46 students in Group 3, four students in Group 4, and seven students in Group 5. We found a significant interaction effect between time and group on students' perceived knowledge transfer, *F*_(9.36, 213)_ = 8.32, *p* <0.001, η^2^ = 0.19. This result suggests that the developmental pattern of perceived knowledge transfer varied across groups of students. Figure 2 shows the developmental pattern of perceived knowledge transfer for each group.

Group 1 accounted for 29% of the whole sample. Students in this group reported moderate levels of perceived knowledge transfer in Stage 1. There was a slight rise in perceived knowledge transfer from Stage 1 to 2; however, it gradually decreased since Stage 2. Group 2 accounted for 14% of the sample. These students demonstrated a high level of perceived knowledge transfer at the beginning of the SL program; however, their perceived knowledge transfer steadily declined for the rest of the program. Group 3 accounted for the largest proportion (46%) of the sample. Perceived knowledge transfer score increased from Stage 1 to 2. Although there was a slight drop in Stage 3, it rebounded and peaked in the last stage of SL. Groups 4 and 5 accounted for 7 and 4% of the sample, respectively. These two groups had similar patterns during Stages 1 and 2. Students had relatively low perceived knowledge transfer scores at first. Then, the scores increased in Stage 2. Dramatic differences between Groups 4 and 5 were observed after Stage 2. For Group 4, there was a steady decline in students' perceived knowledge transfer; in contrast, Group 5's perceived knowledge transfer increased sharply from Stage 2 to 3, and it maintained the same level until the end.

### 3.3. Trends in Students' Perceived Knowledge Transfer Were Associated With Students' Mastery Goal Orientation and Their Perceptions of Psychologically Controlling Behaviors From Their Supervisors

To further understand the differences among students in terms of their perceived knowledge transfer development, we tested the predicting effects of perception of psychological control and mastery goal orientation on group membership (profile). Groups 4 and 5 were excluded from the following analyses as the sample sizes of these two were limited. The results of multinomial logistic regression are shown in Table 1. Students who perceived more psychologically controlling behaviors from their supervisors had higher possibilities of membership in Group 1 relative to Group 3. Students with higher scores of mastery goal orientation presented higher possibilities of membership in Group 3 relative to Group 1.

**Table 1:** The results of multinomial logistic regression.

		**B (SE)**	**95% CI**	**Odds ratio**
Group 1 vs. Group 2^†^	Mastery goal orientation	−0.89 (0.73)	[−2.32, 0.54]	0.41
	Psychological control	0.47 (0.70)	[−0.89, 1.84]	1.61
Group 1 vs. Group 3^†^	Mastery goal orientation	−1.44^*^ (0.59)	[−2.60, −0.29]	0.24
	Psychological control	1.19^*^ (0.58)	[0.05, 2.32]	3.27
Group 2 vs. Group 3^†^	Mastery goal orientation	−0.55 (0.71)	[−1.94, 0.83]	0.57
	Psychological control	0.71 (0.72)	[−0.69, 2.12]	2.04

The groups with

† *serve as baselines in the models*.

* *p <0.05*.

Groups 1 and 3 accounted for 75% of the whole sample. As shown in Figure 2, the major difference in the perceived knowledge transfer patterns between Groups 1 and 3 was observed between Stages 3 and 4. For Group 1, students' perceived knowledge transfer remained steady across the two stages. In contrast, Group 3 demonstrated a marked increase in perceived knowledge transfer from Stage 3 to 4. Compared to Group 1, Group 3 showed a more adaptive trend in perceived knowledge transfer. These findings suggest that college students' perceived knowledge transfer development during a 9-week SL program may be promoted by mastery goal orientation and impeded by perceptions of psychological control.

## 4. Discussion

Although the positive impact of SL on college students' knowledge transfer has been well-established (Markus et al., 1993; Eyler and Giles, 1999; Deeley, 2010; Prentice and Robinson, 2010; Gerholz et al., 2018; Wang et al., 2019b), the developmental characteristics of knowledge transfer as well as the influencing factors have not been investigated. In the current study, we investigated how students' perceived knowledge transfer evolved within the context of a 9-week SL program and examined the impact of mastery goal orientation and perception of psychological control on this process. By providing evidence of the dynamic process and mechanisms of students' perceived knowledge transfer development, the present study contributes to our understanding of how, when, and why the benefits of SL are realized. It directly addresses calls for investigating the underlying mechanisms of how SL enhances student academic outcomes (Eyler, 2000; Furco et al., 2016).

Drawing upon the developmental stages identified in previous studies (Li et al., 2016; Guo et al., 2016b), we divided the 9-week SL program into four stages, namely the confusion and hesitancy stage, the enlightenment and enthusiasm stage, the fluctuation and adjustment stage, and the stabilization and routinization stage. Despite these stages being initially identified to describe the characteristics of student engagement, the changes of perceived knowledge transfer across eight time points demonstrated the same pattern. This is not surprising because the link between engagement and academic success has been consistently demonstrated in traditional learning contexts (Finn and Zimmer, 2012) as well as in SL (Wang et al., 2019b).

On the question of the development pattern of perceived knowledge transfer, we found that students' perceived knowledge transfer in SL did not follow a linear trajectory. Although students' perceived knowledge transfer at the end of SL (i.e., Stage 4) was significantly higher than those at the beginning (i.e., Stage 1), a drop was observed in the middle of SL during Stage 3 (4th to 7th time). The drop in perceived knowledge transfer might be related to the development of the SL activities. After implementing and revising interaction plans several times, students started to establish routines for their SL activities during Stage 3. Rather than setting new goals or designing new interactive activities, students were more likely to make minor modifications to their interaction plans. Although they still used the knowledge they learned from classes to serve children with special needs, students tended to underrate their levels of knowledge transfer as the learning principles that were included in their interaction plans or activities were mostly adopted from previous ones rather than newly added. The changes in SL activities may also explain the rebound in perceived knowledge transfer during Stage 4 (8th time). In the last SL activities, students not only implemented their accustomed interaction activities but also designed new activities to celebrate the end of SL with the recipients. Unlike behavior modifications, a farewell celebration focuses on emotional communication and creating a relaxing atmosphere, which offers students opportunities to apply new knowledge and techniques related to learning sciences.

Another important finding was that the developmental pattern of perceived knowledge transfer in SL varied across students. Although five groups were identified with model-based clustering, 75% of the students belong to Groups 1 and 3. The developmental pattern of perceived knowledge transfer for Group 3 is similar to the one for the whole sample. Despite a slight drop during Stage 3, students' perceived knowledge transfer increased throughout the SL program. Students in Group 1 had a similar developmental pattern as Group 3 between Stages 1 and 3; however, their perceived knowledge transfer did not pick up during the last stage of the program. Compared to Group 1, students in Group 3 demonstrated a more adaptive developmental pattern of perceived knowledge transfer. The variability in college students' perceived knowledge transfer development suggests that teachers should be mindful of students' cognitive and emotional states when implementing SL activities and provide them with distinct interventions.

Drawing from two contemporary motivation theories, we examined the associations of two social-cognitive variables—mastery goal orientation and psychological control—with patterns of perceived knowledge transfer development. The present investigation contributes to our understanding of the social and cognitive factors influencing students' learning development when in SL contexts. The findings showed that students who perceived less psychological control from their supervisors and possessed higher levels of mastery goal orientation had higher possibilities of membership in Group 3. That is, they were more likely to demonstrate adaptive development patterns in perceived knowledge transfer during a 9-week SL program. Mastery goal orientation and psychological control play essential roles in affecting college students' perceived knowledge transfer development and raise the question as to what strategies instructors may use to promote mastery goal orientation and stop being psychologically controlling in the context of SL. Evidence from self-determination theory research suggests a number of approaches, such as providing students with choices, acknowledging students' perspectives, providing meaningful rationales, avoiding controlling language (e.g., “should,” “must,” “have to”), and staying away from salient reward contingencies (Ryan and Deci, 2017). To foster students' mastery goal orientation, the TARGET framework (Ames, 1992) provides instructors with a toolbox of teaching strategies for creating mastery-oriented learning environments, such as focusing attention on students effort, not on abilities, de-emphasizing the negative consequence of making errors, and helping students establish feasible, but challenging goals.

The current study has several limitations we should note. First, the findings are exploratory in that they represent student experiences within a single SL program that was embedded in the course of *Psychology of Learning*. As such, the sample does not represent all fields and potentially over-samples students along gender lines. It would be beneficial to replicate these results in a variety of different types of courses and fields. Second, students' perceived knowledge transfer was assessed with a single-item measure. Although single-item measures generally perform well when gauging a holistic perception (Youngblut and Casper, 1993), as is the case here, a multiple-item measure would be necessary if researchers intend to obtain a better estimate of the construct. Third, it is intriguing that mastery goal orientation and perception of psychological control were associated with patterns of perceived knowledge transfer development; however, there may be alternative influential factors based on other theoretical frameworks that future research needs to explore. Moreover, the findings about the changes in perceived knowledge transfer need to be interpreted with caution, as we do not have a comparison group showing how students' perceived knowledge transfer evolves in a regular lecture-based learning context. Further quasi-experimental investigations are needed to determine the impact of SL on the development of perceived knowledge transfer.

## Data Availability Statement

The raw data supporting the conclusions of this article will be made available by the authors, without undue reservation.

## Ethics Statement

The studies involving human participants were reviewed and approved by Beijing Normal University. The patients/participants provided their written informed consent to participate in this study.

## Author Contributions

CW and MY designed the study. CW, FG, YL, and MY collected the data. CW and WY formulated the hypotheses. CW performed the statistical analyses and drafted the manuscript. CW, WY, FG, YL, and MY revised and edited the manuscript. All authors contributed to the article and approved the submitted version.

## Conflict of Interest

The authors declare that the research was conducted in the absence of any commercial or financial relationships that could be construed as a potential conflict of interest.
